# Evolution of *Pseudomonas aeruginosa* toward higher fitness under standard laboratory conditions

**DOI:** 10.1038/s41396-020-00841-6

**Published:** 2020-12-03

**Authors:** Igor Grekov, Janne Gesine Thöming, Adrian Kordes, Susanne Häussler

**Affiliations:** 1 Department of Molecular Bacteriology, Helmholtz Centre for Infection Research, Braunschweig, Germany; 2 Institute of Molecular Bacteriology, TWINCORE Centre for Experimental and Clinical Infection Research, Hannover, Germany; 3 Department of Clinical Microbiology, Copenhagen University Hospital - Rigshospitalet, Copenhagen, Denmark; 4 Cluster of Excellence RESIST (EXC 2155), Hannover Medical School, Hannover, Germany

**Keywords:** Molecular evolution, Bacterial genetics, Functional genomics, Transcriptomics, Biofilms

## Abstract

Identifying genetic factors that contribute to the evolution of adaptive phenotypes in pathogenic bacteria is key to understanding the establishment of infectious diseases. In this study, we performed mutation accumulation experiments to record the frequency of mutations and their effect on fitness in hypermutator strains of the environmental bacterium *Pseudomonas aeruginosa* in comparison to the host-niche-adapted *Salmonella enterica*. We demonstrate that *P. aeruginosa*, but not *S. enterica*, hypermutators evolve toward higher fitness under planktonic conditions. Adaptation to increased growth performance was accompanied by a reversible perturbing of the local genetic context of membrane and cell wall biosynthesis genes. Furthermore, we observed a fine-tuning of complex regulatory circuits involving multiple di-guanylate modulating enzymes that regulate the transition between fast growing planktonic and sessile biofilm-associated lifestyles. The redundancy and local specificity of the di-guanylate signaling pathways seem to allow a convergent shift toward increased growth performance across niche-adapted clonal *P. aeruginosa* lineages, which is accompanied by a pronounced heterogeneity of their motility, virulence, and biofilm phenotypes.

## Introduction

*Pseudomonas aeruginosa* is an environmental generalist and an opportunistic pathogen capable of infecting a wide variety of hosts ranging from plants to humans [1, 2]. Morphological, physiological, and behavioral changes that allow an organism to adjust to changing conditions arise as the result of two key mechanisms: phenotypic plasticity and natural selection [3]. Phenotypic plasticity is conferred by regulatory circuits that coordinate the perception of different signals, integrate them, and respond by altering gene transcription, protein translation, and protein activity. Natural selection acts upon a genetically diverse population and promotes the maintenance of alleles that are beneficial and the loss of alleles that are deleterious in a given environment. In addition to the acquisition of mutations and new genes via horizontal gene transfer, phase variation can produce genetic diversity in changing environments. This reversible process involves the variation of gene expression via the stochastic on–off switching of gene activity [4], mediated by insertions and deletions in simple sequence repeats [5–8], rearrangements in gene or promoter regions [9–11] or by epigenetic mechanisms [12].

The genomes of *P. aeruginosa* strains are large (5–7 Mbp) and encode a variety of regulatory circuits. These provide a remarkable phenotypic plasticity and a high adaptation potential to the opportunistic pathogen. For the successful colonizing of new habitats, *P. aeruginosa* must maintain this adaptation potential even under constant conditions. However, as it has been observed for various bacteria and yeasts, constant conditions favor the genetic selection of niche specialists, which have a higher fitness, but show fitness decay in other environments [13]. The trade-offs between higher fitness in one, but lower fitness in other environments result from the accumulation of mutations, which are beneficial or neutral under the selective conditions, but deleterious in other environments [13–18].

In this study, we performed mutation accumulation (MA) experiments in the environmental bacterium *P. aeruginosa* and recorded the type and frequency of mutations as well as their effect on bacterial fitness as compared to *S. enterica*. We used DNA repair-deficient bacterial strains characterized with ~100 times higher mutation rates in order to accelerate MA. Each of the species was continuously cultivated in the exponential phase under weak and strong bottlenecking conditions. In the context of experimental evolution, strong bottlenecking and a lower size of the effective population are thought to promote fixation of random mutations and, since most mutations are deleterious, lead to a loss of fitness. Conversely, weak bottlenecking enables natural selection and is more favorable to the fixation of beneficial and elimination of harmful mutations [19, 20]. Of note, the use of hypermutators in this study can undermine adaptive evolution, because a hypermutator with a beneficial mutation has a higher chance to acquire a detrimental second mutation, so that the evolutional behavior will differ to non-mutator populations. We demonstrate that under weak bottlenecking, *P. aeruginosa*, but not *S. enterica* hypermutator strains adapt to planktonic rich medium conditions by evolving increased growth performance. We show that the increase in fitness in *P. aeruginosa* was associated with an increased rate of phase variation events in the promoter regions of genes involved in membrane and cell wall biogenesis. Furthermore, the fine-tuning of cyclic di-guanylate (c-di-GMP) signaling-governed pathways was observed, indicating that *P. aeruginosa* adapts to lifestyles between planktonic and biofilm growth modes.

## Materials and methods

### Strains and culture conditions

Mismatch-repair-deficient mutants of *P. aeruginosa* and *S. enterica* were taken from a PA14 transposon insertion library [21] and provided by Prof. M. Erhardt (the *mutS* gene was replaced with an Frt-Kanamycin-Frt cassette in the *S. enterica* serovar Typhimurium LT2 background), respectively. Bacterial cultures were grown in 96-well plates with continuous shaking at 180 rpm. To synchronize the growth of the studied species, *P. aeruginosa* and *S. enterica* cultures were grown at 37 °C in LB medium (1% tryptone, 0.5% yeast extract, 0.75% NaCl) and at 30 °C in LB medium supplemented with 0.2% glucose, respectively (Fig. S1).

### Experimental evolution

**Fig. 1:**
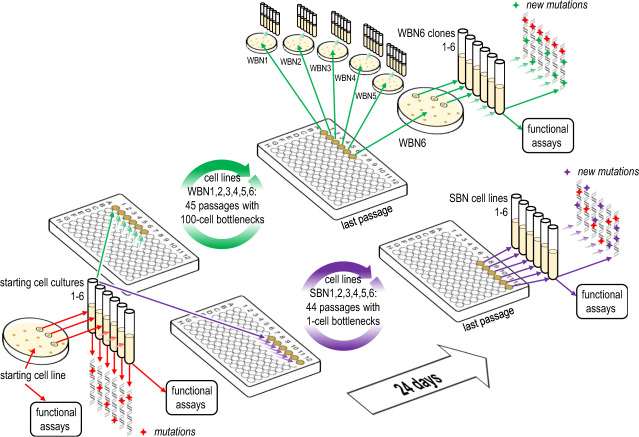
Schematic overview of the mutation accumulation experiment. *P. aeruginosa mutS*::Tn (the starting cell line) was streaked on LB agar. Six colonies were picked and used to inoculate the overnight cultures (the starting cultures). Each of the starting cultures was used to inoculate a weak bottleneck (WBN) cell line with 100 cells and a strong bottleneck (SBN) cell line with one cell. WBN and SBN cell lines were propagated for 24 days. Six single-colony-derived clones for each WBN cell line were saved in liquid nitrogen. Subsequently, whole-genome sequencing of cryo-stocked starting cultures, last passage of the SBN cell lines and the six WBN clones per each WBN cell line was performed. We also performed an analogous mutation accumulation experiment with *S. enterica* LT2 ∆*mutS* using 250 cells for passaging WBN cell lines.

Starting cell lines of *P. aeruginosa* PA14 *mutS*::Tn and *S. enterica* LT2 ∆*mutS* were streaked out on LB plates, and six randomly selected colonies were used to inoculate starting cultures (Fig. 1). After 10.5 h of growth, *P. aeruginosa* strong bottleneck (SBN) and weak bottleneck (WBN) cultures were diluted to 1 and 100 cells per 100 μl, respectively, and *S. enterica* cultures were diluted to 1 (SBN) and 250 (WBN) cells per 100 μl (see Supplementary Note S1). New WBN and SBN cultures were prepared every 12 h. All the cultures were propagated for 24 days. At the end of the MA experiment, SBN cultures were used to prepare 4 ml overnight cultures in LB medium or LB + 0.2% glucose, which were subsequently aliquoted and stored at −70 °C. The WBN cultures were streaked on LB agar plates to isolate single colonies. Nine colonies were randomly selected per plate and subsequently used to prepare 4 ml overnight cultures in LB medium or LB + 0.2% glucose. Following overnight growth, aliquots from the WBN clonal cultures were stored at −70 °C.

### Genome sequencing and mutation analysis

For whole-genome sequencing, genomic DNA was isolated from, in total, 83 planktonic overnight cultures using the DNeasy Blood & Tissue kit (Qiagen, Hilden, Germany) and fragmented using the S2/E210 Focused-ultrasonicator (Covaris, Woburn, MA) to achieve 400-nt-long fragments. Libraries were prepared using the NEBNext Ultra DNA Library Prep Kit for Illumina (NEB, Ipswich, MA) according to the manufacturer’s instructions. The libraries were sequenced using Illumina MiSeq (2 × 250-bp paired-ended reads). Reads were mapped to PA14 reference genome using Burrow–Wheeler Aligner [22], re-mapped with Stampy [23], and subsequently processed using SAMtools [24] and custom scripts written in Python (Python Software Foundation, https://www.python.org/). The sequencing coverage was analyzed using SAMtools and was determined to be on average 32×. After quality filtering of specific nucleotide variations, mutations were checked using Integrative Genomics Viewer (v.2.3.98) [25]. The analysis of the types and topology of mutations was performed using custom R scripts (for details concerning indel analysis, see Supplementary Note S2).

### Transcriptome sequencing and analysis

RNA sequencing was performed for three randomly selected clonal isolates from each WBN cell line. RNA was extracted from the logarithmic stage cultures of the selected clones and further processed as described in the protocol in Supplementary Note S3. The cDNA libraries yielded by the protocol were subsequently sequenced with an Illumina HiSeq 2500 in single end mode. Reads were mapped to PA14 reference genome with Stampy, and the data were processed using custom Perl (The Perl Foundation, https://www.perl.org/) and C shell scripts. Differential gene expression was estimated with the help of custom R scripts utilizing DESeq2 [26], tidyverse, pheatmap, and RColorBrewer packages.

### Phenotypic assays

Fitness was analyzed by determining the optical density of the cultures throughout the growth phase. Swimming assays were performed as previously described [27]. Virulence was determined using a *Galleria mellonella* (greater wax moth) infection model [28]. Biofilm thickness and volume were evaluated using automated confocal microscopy [29, 30]. In addition, biofilm-forming capacity of the evolved strains was assessed based on their ability to adhere to a polyvinyl chloride surface, as measured by the crystal violet assay [31]. The detailed protocols are described in Supplementary Notes S4–S8.

## Results

### MA experiment

We performed serial passaging of a mismatch-repair-deficient *P. aeruginosa* mutant through defined weak bottlenecks (WBNs) in standard laboratory conditions (Fig. 1). Five independent WBN cell lines were continuously propagated in the exponential growth phase in LB medium for 24 days. WBN cell lines were passed through 45 bottlenecks by transferring 100 cells into fresh culture medium every 12 h. We estimate that WBN cell lines were grown for ~870 generations with 19 generations between each bottleneck. In order to evaluate the spontaneous mutation rate, and to analyze the influence of randomly accumulated mutations on bacterial fitness, we included a control experiment. In this experiment, selection and adaptation were prevented by passaging *P. aeruginosa* through SBNs. Six independent SBN cell lines were passaged through 44 bottlenecks by transferring a single cell into fresh medium over the course of 24 days. The SBN cell lines were grown for around 950 generations, resulting in ~21 generations between passages.

### *P. aeruginosa* adapts to exponential LB growth conditions and gains fitness

**Fig. 2:**
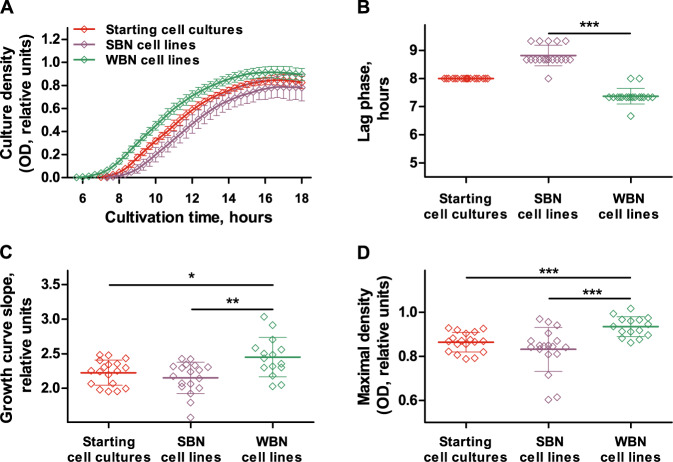
Growth characteristics of the evolved *P. aeruginosa* clones. The growth of six *P. aeruginosa* starting cultures, six SBN cell lines, and the average growth of 30 clones from overall five WBN cell lines was recorded in standard LB medium (three independent experiments, technical triplicates) (**A**). The lag phase (**B**) and the growth rate (**C**) were calculated from growth curves using linear regression. **D** depicts the maximal ODs. Graph **A** shows average values and standard deviation, graphs **B**–**D** show individual values, average values, and standard deviation. The differences between growth parameters were calculated using the Mann–Whitney test. * corresponds to *p* < 0.05, ** corresponds to *p* < 0.01, and *** corresponds to *p* < 0.001.

To elucidate whether the five evolved *P. aeruginosa* WBN cell lines adapted to the cultivation conditions of the MA experiment, each WBN cell line was streaked out on LB agar, six colonies per cell line were isolated and their growth behavior in LB was compared with the growth behavior of the starting cultures (Fig. 1). Optical density (OD_600_) measurements demonstrated that evolved *P. aeruginosa* WBN clones developed a shortened lag phase (Fig. 2A, B), grew faster in the exponential phase (Fig. 2C), and reached higher maximal OD_600_ values (Fig. 2D). We confirmed for three randomly picked WBN clones that they evolved superior growth behavior by determining colony-forming units/ml (Fig. S2). In addition, we showed that the cells of the starting line and one of the randomly selected WBN clone do not differ in size (Fig. S3 and Supplementary Note S9), corroborating that higher OD_600_ values in evolved clones reflect higher cell numbers. Regression analysis demonstrated a positive correlation between growth rate and maximal cell density in the clones from four out of five WBN cell lines (Fig. S4). Taken together, *P. aeruginosa* WBN cell lines adapted to the conditions of the MA experiment by evolving shorter lag phase, faster growth rates, and increased growth yield.

In a control experiment, we analyzed the fitness of *P. aeruginosa* SBN cell lines. Under extreme bottlenecking conditions, genetic drift dominates evolution as the influence of natural selection is reduced and MA occurs mainly by chance [13]. In accordance with previous observations that random MA in SBN cultures results in a decrease in fitness [32], the analysis of growth behavior of the evolved *P. aeruginosa* SBN cell lines revealed no signs of adaptation to the LB cultivation conditions. Instead, the evolved SBN cell lines had a growth disadvantage. This was characterized by a longer lag phase and a slower growth during the first hours of cultivation (Fig. 2A, B).

We then investigated whether the fitness advantage of the *P. aeruginosa* WBN clones in LB produced trade-offs between growth in LB and in chemically defined nutrient-rich BM2 medium. We observed that the phenotypes of the studied clones ranged from the complete loss of ability to grow in BM2 to even increased growth rates, shortened lag phase, and higher maximal density in this medium (Fig. S5). Thus, the adaptation of the WBN clones to growth in LB did not necessarily produce fitness trade-offs in BM2 medium. Furthermore, the variability in BM2 growth parameters among the different clones indicates that they reached higher fitness in LB via different adaptive mechanisms.

### *S. enterica* did not gain fitness upon sustained growth in LB

**Fig. 3:**
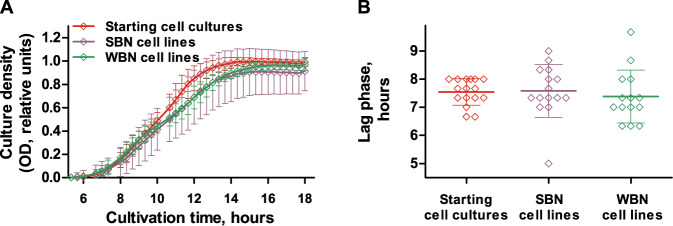
Growth characteristics of the evolved *S. enterica* clones. The growth of six *S. enterica* starting cultures, six SBN cell lines, and the average growth of 30 clones from overall five WBN cell lines was recorded in LB medium + 0.2% glucose (three independent experiments, technical triplicates) (**A**). The lag phase of *S. enterica* was calculated from their growth curves using linear regression (**B**). Graph **A** shows average values and standard deviation and graph **B** shows individual values, average values, and standard deviation. No statistically significant differences between lag phases were identified as calculated using the Mann–Whitney test.

In an analogous MA experiment, WBN and SBN cell lines of mismatch-repair-deficient *S. enterica* were cultivated for 24 days in logarithmic phase. During this time, *S. enterica* cultures were passed through 45 bottlenecks. WBN cultures of *S. enterica* were grown for ~840 generations, and SBN cultures of *S. enterica* were grown for ~1000 generations, equivalent to 18 and 22 generations between bottlenecks, respectively. The subsequent evaluation of the growth behavior of the evolved *S. enterica* WBN and SBN cell lines revealed considerable differences to that of *P. aeruginosa*. While we observed a consistent increase in fitness of the *P. aeruginosa* WBN clones, the *S. enterica* WBN clones showed no significant increase in fitness (Fig. 3A, B). In addition, unlike the *P. aeruginosa* SBN cell lines, the *S. enterica* SBN cell lines exhibited no significant fitness loss.

### Intergenic indels show strong selection patterns in *P. aeruginosa* but not in *S. enterica*

For the identification of accumulated mutations, the WBN and SBN cell lines were subjected to whole-genome sequencing at the end of the MA experiments (Fig. 1). In addition, we sequenced the genomic DNA of the six starting cultures used to inoculate our WBN and SBN cell lines. The evolved *P. aeruginosa* WBN and SBN cell lines accumulated 123.2 ± 13.3 and 146.0 ± 15.7 mutations, respectively, which corresponded to 22 ± 2 and 23 ± 2 mutations/Mbp/10^3^ generations. The evolved *S. enterica* WBN and SBN cell lines acquired 69.3 ± 8.4 and 80.7 ± 9.3 mutations, respectively, which corresponded to 17 ± 1 and 17 ± 2 mutations/Mbp/10^3^ generations and thus closely resembled the mutation rates in *P. aeruginosa*. The rates were comparable to those reported previously in *P. aeruginosa* and *Escherichia coli* MA experiments using DNA repair-deficient strains [32, 33]. The most prevalent type of mutations were nucleotide substitutions (Fig. S6). As was to be expected in the hypermutator strains [32], the increase in mutation rates resulted primarily from the increase in the rate of transitions (Fig. S7). These accounted for more than 80% of all accumulated mutations and more than 95% of accumulated nucleotide substitutions in the WBN and SBN lines of *P. aeruginosa* and *S. enterica*.

**Fig. 4:**
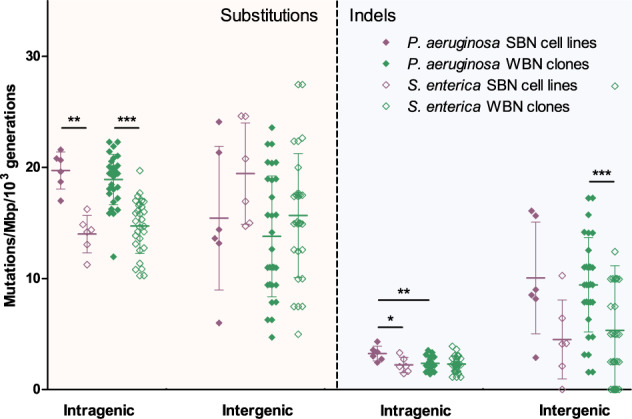
The rates of nucleotide substitutions and indels according to location in coding and intergenic regions. The individual values, the mean values, and the standard deviations for each class of mutations and each experimental group are shown. All the mutation rates are normalized to the number of generations in the corresponding experimental group. The rates of nucleotide substitutions and indels in the coding regions are normalized to the total length of coding regions in the corresponding species. The rates of nucleotide substitutions and indels in the intergenic regions are normalized to the total length of intergenic regions in the corresponding species. The differences between growth parameters were calculated using the Mann–Whitney test. * corresponds to *p* < 0.05, ** corresponds to *p* < 0.01, and *** corresponds to *p* < 0.001.

To identify mutation patterns that reflect the *P. aeruginosa* fitness increase in the WBN and the fitness decay in the SBN cell lines, we performed a more in-depth mutation analysis. The rate of intragenic as well as intergenic nucleotide substitutions did not differ between WBN and SBN cell lines of *P. aeruginosa* or *S. enterica*. However, the intragenic mutation rate was ~25% higher in *P. aeruginosa* as compared to *S. enterica* (*p* < 0.001, Mann–Whitney test) (Fig. 4). Among the genes that harbored intragenic nucleotide substitutions, we did not identify an enrichment of any Clusters of Orthologous Groups (COG) functional category in the WBN or SBN cell lines, in both *P. aeruginosa* and *S. enterica*. Taken together, despite the overall higher single nucleotide polymorphism (SNP) rates in *P. aeruginosa*, no differences in nucleotide substitution rates between WBN and SBN cell lines in either of the species were detected, indicating that there was no clear selection pattern for SNPs in the various bottlenecking conditions of our MA experiment.

We then focused our analysis on indels. We found indels mostly in DNA homopolymeric tracts, which are the simplest of simple sequence repeats, and which are considered to be mutation hotspots [34]. The rate of intragenic indels did not differ between *S. enterica* WBN and SBN cell lines, and the rate of intragenic indels in *P. aeruginosa* WBN cell lines was comparable to that of the *S. enterica* WBN cell lines. However, the rate of intragenic indels in *P. aeruginosa* SBN cell lines was ~50% higher as compared to the *P. aeruginosa* WBN cell lines (*p* < 0.01, Mann–Whitney test) (Fig. 4). Intragenic indels frequently cause frameshifts, and frameshift variants tend to be more deleterious. The fact that we found less indels in the intragenic regions of the WBN cell lines indicates that they were subject to purifying selection in *P. aeruginosa*. At the same time, the intragenic indels in *P. aeruginosa* WBN cell lines showed no enrichment in any functional gene category. Thus, the indels that were found, did not seem to be under positive selection.

In the intergenic regions, the rate of indels did not differ between WBN and SBN cell lines of the same species (Fig. 4). However, the rate of intergenic indels was twice as high in *P. aeruginosa* as compared to *S. enterica* (*p* < 0.001 for WBN cell lines and *p* = 0.09 (nonsignificant) in SBN cell lines, Mann–Whitney test). Interestingly, we found an enrichment in intergenic indels that preceded genes of the functional COG category “cell wall/membrane/envelope biogenesis” (*p* < 0.01, chi-square test) exclusively in the *P. aeruginosa* WBN cell lines, indicating that there was a positive selection for the respective indels.

To further confirm that the indels in the intergenic homopolymeric tracts are under positive selection in the *P. aeruginosa* WBN clones, we re-analyzed the previously recorded genome sequences of 414 *P. aeruginosa* clinical isolates [35]. Thirty out of 45 *P. aeruginosa* indels that were exclusively found in WBN cell lines were also found in at least one and up to 213 clinical isolates. At the same time only 9 out of 22 indels that recorded exclusively in upstream gene regions in *P. aeruginosa* SBN cell lines were found in at least one clinical isolate. The significantly higher rate of intragenic WBN indels that were found in the clinical isolates (*p* < 0.05, chi-square test) implies that the intergenic indels in the *P. aeruginosa* WBN cell lines are subject to positive selection also in *P. aeruginosa* clinical isolates, probably, due to their effect on the transcription and/or translation of downstream genes. Since a majority of the indels are within homopolymeric tracts, phase variation might facilitate adaptation to rapidly varying environments without the requirement for random mutations.

### Gene expression analysis in *P. aeruginosa* WBN cell lines showed convergent and divergent gene expression changes

**Fig. 5:**
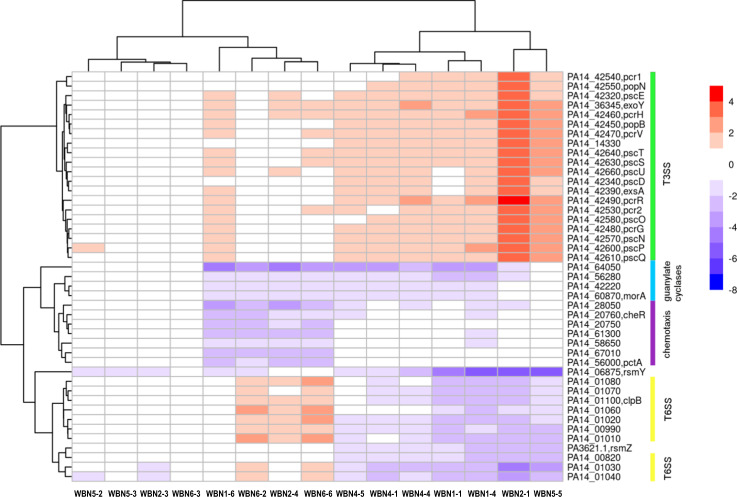
Heat map depicting the selected gene expression changes of the evolved clones. RNA sequencing was performed for three randomly selected clonal isolates from each WBN cell line. Gene expression changes in 15 WBN clones as compared to the starting cell line are indicated as a color code. Selected genes that were found to be differentially regulated in at least four WBN clones and that belonged to the enriched COG categories (“signal transduction,” “cell motility,” and “intracellular trafficking, secretion, and vesicular transport”) are shown.

To obtain a deeper understanding of how the genetic changes in the *P. aeruginosa* WBN clones are translated into fitness changes, we randomly selected three isolates from each of the five WBN cell lines and compared the gene expression profile of these 15 evolved WBN clones with that of the starting cell line (Table S1). The expression profiles of the three clones derived from each of the five WBN cell lines typically clustered together, reflecting their common origin (Fig. 5). Each clone also exhibited a number of unique gene expression changes. However, there were also transcriptional changes that were shared by the clonal isolates from different WBN cell lines. We found a functional enrichment of differentially expressed genes, which were assigned to the COG categories “signal transduction,” “cell motility,” and “intracellular trafficking, secretion, and vesicular transport” and to the PseudoCAP categories “cell wall/lipopolysaccharides/capsule,” “chemotaxis,” “motility and attachment,” “protein secretion/export apparatus” and “secreted factors (toxins, enzymes, alginate).” It was shown that the majority of WBN clones (12 of 15) exhibited downregulation of *rsmY*, which regulates the transition from planktonic lifestyle to biofilm lifestyle, and seven exhibited an additional downregulation of *rsmZ* (Fig. 5), which acts in synergy with *rsmY*. Six of the seven *rsmZ/rsmY*-low expressing clones, and one of the *rsmY*-low expressing clones, showed a significant upregulation of type-III secretion system (T3SS) genes, including PA14_14330 (*spcS* ortholog), *exoY, pscBCDE, exsABC*, PA14_42380 (*exsD* ortholog), PA14_42410 (*exsE* ortholog), *popDBN, pcrDGHRV, pcr1-4* orthologs, *pscNOPQRTU,* and PA14_42630. The *rsmZ/rsmY*-downregulating clones also showed lower expression of type-VI secretion system (T6SS) genes (*tagQ1R1S1T1, ppkA, pppA, tagF1J1, icmF1*, *tssA1, tssE1F1G1, tssJ1K1L1*, PA14_00970 (*fha* ortholog), *fha1, hsiB1, hsiC1, hcp1, clpV1*) (Fig. 5). Four of the clones, which did not downregulate *rsmZ*, downregulated at least seven genes involved in chemotaxis signaling (*pctA*, PA14_20750 (*cheW* ortholog), PA14_20760 (*cheR* ortholog), and chemotaxis transducers PA14_28050, PA14_58650, PA14_61300, and PA14_67010) (Fig. 5). Interestingly, only one of four clones with downregulated expression of chemotaxis-related genes showed a minor upregulation of the T3SS, while the other three clones showed upregulation of the T6SS. Last, the downregulation of four genes known to encode di-guanylate cyclases (*morA*, PA14_42220 (*mucR* ortholog), PA14_64050 (*pleD* ortholog), and PA14_56280 (*sadC* ortholog)) were found in two thirds of the clones (9 out of 15). Changes in expression of *rsmZ, rsmY*, and genes encoding T3SS, T6SS, and di-guanylate cyclases are well known to be linked to the switch between motile and sessile lifestyles [36–43]. This prompted us to investigate if the aforementioned alterations in gene expression result in coherent changes in motility, virulence, and biofilm formation.

### Swimming motility, virulence, and biofilm formation were highly variable between individual evolved clones

**Fig. 6:**
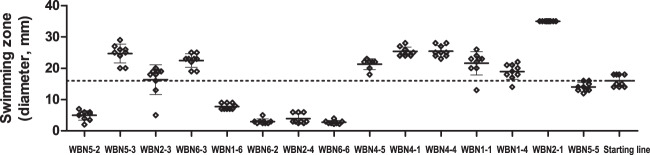
Motility phenotypes of the evolved clones. Swimming motility of the starting cell line and of the 15 WBN clones with recorded transcriptional profiles was estimated based on the diameter of the swimming zones on BM2 agar plates. The graph shows individual values, average values, and standard deviation (three independent experiments with three technical replicates). To facilitate the comparison of phenotypic data and transcriptional profiles, the WBN clones are arranged along *x* axis in the same order as they are arranged by DESeq2 clustering analysis.

We observed a high variation in swimming motility between individual WBN clones (Fig. 6). Their phenotypes ranged from the complete absence of swimming motility to much higher motility than observed for the starting cell line. The differences were high even between WBN clones that belonged to the same cell line. As expected, all four WBN clones with low expression of chemotaxis genes showed low swimming motility.

**Fig. 7:**
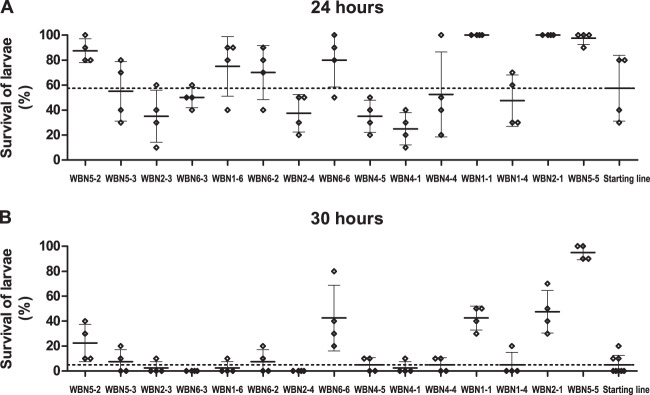
Pathogenicity phenotypes of the evolved clones in the *Galleria mellonella* assay. Survival rates of *G. mellonella* after 24 h (**A**) and 30 h (**B**) following infection of the larvae with the starting cell line and the 15 WBN clones with recorded transcriptional profiles are shown. PBS was used as negative control. The graph shows individual values, average values, and standard deviation (in at least two independent experiments with two technical replicates, minimally 40 larvae per clone were infected.). To facilitate the comparison of phenotypic data and transcriptional profiles, the WBN clones are arranged along *x* axis in the same order as they are arranged by DESeq2 clustering analysis.

**Fig. 8:**
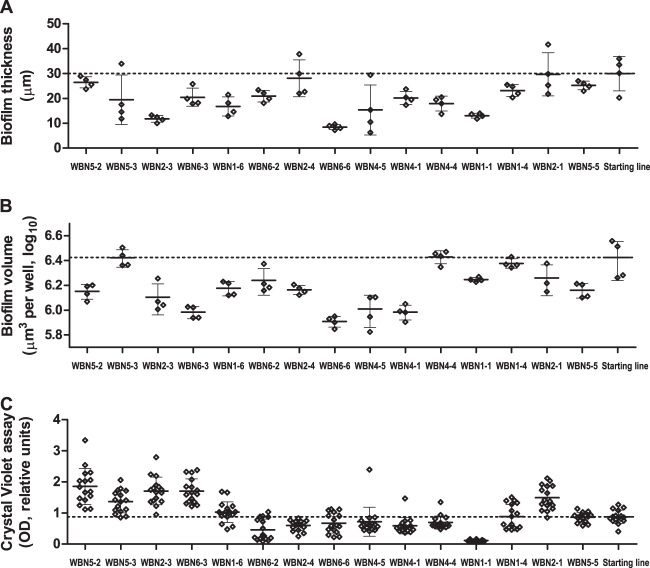
Biofilm phenotypes of the evolved clones. Biofilm thickness (**A**) and biofilm volume (**B**) was recorded in two independent experiments, two technical replicates using automated confocal microscopy. Biofilm-forming capacity according to the crystal violet assay (**C**) was recorded in two independent experiments with eight technical replicates. All the graphs show individual and average values and standard deviation. To facilitate the comparison of phenotypic data and transcriptional profiles, the WBN clones are arranged along *x* axis in the same order as they are arranged by DESeq2 clustering analysis.

Similarly to swimming motility, virulence of the evolved WBN clones, as determined by the use of the *G. mellonella* model, showed extreme variability with phenotypes ranging from almost avirulent to more virulent than the starting cell line (Fig. 7). Moreover, virulence also differed between clones derived from the same WBN cell line. Of note, virulence of WBN clones did not correlate with the expression of the T3SS. The estimation of biofilm production using confocal microscopy and the crystal violet assay demonstrated that the WBN clones had variable phenotypes (Figs. 8A–C and S8) but did not surpass the starting cell line according to biofilm thickness and volume.

## Discussion

*P. aeruginosa* has become a model organism to study experimental evolution and adaptation to diverse and challenging conditions, including adaptation to various carbon sources [44], antibiotic pressure [45], biofilms [46], and conditions encountered during an infection process [47–49]. In this study, we subjected hypermutator strains of *P. aeruginosa* to continuous exponential phase cultivation in rich LB medium under SBN and WBN regimens. While the fitness of *P. aeruginosa* SBN cell lines consistently decayed, competition, enabled by passaging *P. aeruginosa* WBN cell lines through 100-cell bottlenecks, led to the selection of *P. aeruginosa* clones with increased fitness in all five independently propagated WBN cell lines. Growth parameters that were under selective pressure showed improvement in almost all evolved *P. aeruginosa* WBN clones. Moreover, roughly half of the *P. aeruginosa* WBN clones developed higher cell densities in stationary phase, despite a lack of selection for this trait. In the majority of WBN clones, we observed a positive correlation between the growth rate and yield, which distinguished our results from the previous observations of a trade-off between growth rate and growth yield in bacteria characterized by faster growth [50–56]. Nevertheless, in one of the lines (WBN2 cell line) there was evidence for a growth rate/yield trade-off as predicted previously [57].

In BM2, an alternative medium, approximately half of WBN clones retained their full growth advantage. Thus, though adaptation of *P. aeruginosa* to one condition produced fitness trade-offs in other conditions, adaptation of *P. aeruginosa* to one condition also led to simultaneous improvement of fitness in selected alternative conditions as it was previously shown in a different range of bacterial species [13, 15, 58].

Furthermore, our finding of varying growth curve changes in LB and in BM2 medium indicates that *P. aeruginosa* WBN clones acquired fitness via different and independent mechanisms. Of note, passaging *S. enterica* in LB did not result in a change of fitness under any of the cultivation regimens even though the numbers of mutations accumulated by *S. enterica* and *P. aeruginosa* were comparable. Further research would be needed to establish which traits account for different fitness changes in the studied species.

Our finding, and that of a previous study, which reported no increase of growth rates of *S. enterica* following serial propagation in LB medium [59], seems to be contrasting the results of a long-term evolution experiment, in which *E. coli* evolved a shorter lag phase and higher growth rates [60]. However, the experiment was performed in minimal medium with glucose as the sole carbon source, and *E. coli* adapted by increasing glucose uptake [61].

Closer inspection of the mutation profiles of the evolved *P. aeruginosa* and *S. enterica* hypermutator strains revealed that transitions accounted for more than 95% of the nucleotide substitutions and more than 80% of total accumulated mutations. This is in line with previous reports on hypermutator mutation profiles [32, 62]. Despite the fact that most mutations are weakly deleterious [63], SNPs did not seem to be subject of purifying selection in our experimental conditions. According to the drift-barrier hypothesis, weak purifying selection may indicate that the disadvantage of most nucleotide substitutions is so small that the selection against them is insufficient to prevent their fixation [64]. Furthermore, we did not observe an enrichment of SNPs in any functional gene category in *P. aeruginosa* WBN cell lines, which exhibited increased fitness. Thus, on the genome scale, SNPs did not show evidence of positive selection. However, one cannot exclude that the fitness increase in each of WBN clones is at least partially driven by very few strongly beneficial SNPs in genes from different functional categories.

In contrast to SNPs, the indels, the majority of which were found in DNA homopolymeric tracts, displayed clear patterns of natural selection. We found a lower frequency of indels within the coding sequences in *P. aeruginosa* WBN cell lines. Under the assumption that those indels frequently cause frameshifts and thus negatively affect the gene function, their higher frequency in the SBN cell lines might explain the loss of fitness, while their lower frequency in the WBN cell lines might be the result of purifying selection. Unlike the intragenic indels, the intergenic indels were not counter-selected and became fixed in *P. aeruginosa* WBN cell lines. Homopolymeric tract-mediated phase variation has been identified as an adaptive mechanism in a wide range of bacteria [5, 34]. Interestingly, intergenic indels that were exclusively found in *P. aeruginosa* WBN cell lines were enriched in the upstream regions of the genes involved in membrane and cell wall biogenesis, implicating that they were positively selected. Most notably, a large part of intergenic indels encountered in the adapted WBN cell lines, but not in maladapted SBN cell lines, could also be found in a collection of more than 400 clinical *P. aeruginosa* isolates. Our results suggest that, in *P. aeruginosa*, indels in intergenic homopolymeric tracts promote adaptation both, to our experimental conditions and to the environment of the human host. The data are also supported by a recent study, which highlighted the importance of mutations in the intergenic regions for the adaptation of *P. aeruginosa* to the conditions of cystic fibrosis lungs [65]. We suggest that changes in the length of intergenic homopolymeric tracts mediate phase variation, giving rise to a plethora of phenotypically distinct individuals, which may have advantages in colonizing new niches or in coping with adverse conditions [4]. Since efficient adaptation seemed to be based on the positive selection of intergenic indels in our experimental setup, and since indels are not known to be enriched in hypermutator strains, our data also imply that indels in intergenic homopolymeric tracts might not only promote adaptation of *P. aeruginosa* hypermutator but also wild-type populations.

In order to elucidate whether there is another common theme associated with *P. aeruginosa* adaptation to fast growth, we performed transcriptional profiling. Indeed, we found transcriptional changes that were shared between *P. aeruginosa* WBN clones even if they originated from independently evolved cell lines. These results indicate that independent groups of adapting clones followed partly convergent evolutional paths, which has been previously shown both for *P. aeruginosa* [47, 49] and closely related *P. fluorescens* [66]. Common transcriptional changes mainly affected signaling, motility, secretion, and virulence factor production. More specifically, we found a group of clones that exhibited a decreased expression of *rsmZ* and *rsmY*, associated with an increased expression of the T3SS and decreased expression of the T6SS, and another group of clones with a decreased expression of chemotaxis transducers and an induced T6SS. Notably, the trade-off between chemotaxis, motility, and the rapid growth has been described as typical in various bacterial species [67–70]. Also, two thirds of all the clones showed a decreased expression of cyclases that produce the intracellular signaling molecule c-di-GMP.

Low levels of c-di-GMP and low transcription of *rsmZ* and *rsmY* are associated with planktonic lifestyles, characterized by fast growth, high swimming motility, and high virulence [36–43], whereas high levels of c-di-GMP contribute to the switch from planktonic to biofilm lifestyles [40, 71, 72]. Many cyclases increase cellular pools of c-di-GMP locally, thereby impacting selected downstream effector molecules. As a result, the impact of the activity of different cyclases on motility, biofilm, and virulence phenotypes can vary extensively [40, 73–77] e.g., whereas *gcbA*, a PA14_64050 homolog, was shown to impact motility, surface attachment, and biofilm dispersal, but not biofilm formation [78, 79], *morA* showed strain-dependent effects on motility [80, 81] and biofilm formation [77, 81].

The results of the transcriptional analysis and phenotypic assays imply that, in *P. aeruginosa*, adapted clones occupy a large space between the two extreme modes of growth—rapid planktonic growth associated with high virulence and slow biofilm-associated growth with reduced motility and virulence. It seems that the phenotypes of the individual evolved clones are impacted by the variable expression of small regulatory rRNAs, such as *rsmY* and *rsmZ*. In addition, the evolved phenotypes could be the result of an adaptation of the activity of different c-di-GMP modifying enzymes (>45 can be found in *P. aeruginosa* PA14 [77]), which modulate local c-di-GMP pools with different outcomes for motility and virulence. The overall redundancy and local specificity of these regulatory pathways of *P. aeruginosa* allow a convergent shift from the biofilm to planktonic lifestyle, beneficial under the experimental conditions, whereas individual evolved clones still display divergent biofilm, virulence, and motility phenotypes. It must, however, be considered that in our experimental setup an extensive diversification might have resulted from using a hypermutator strain of *P. aeruginosa*. Nevertheless, rapid diversification in nutritionally or structurally complex environments has been demonstrated before for mutator as well as non-mutator *P. aeruginosa* populations [46, 48]. A bet-hedging strategy could ensure that, on the population level, *P. aeruginosa* maintains its versatility and preserves high adaptive potential.

In conclusion, we demonstrated that the WBN clones of the hypermutator *P. aeruginosa* strain evolved higher fitness in LB through a multitude of different pathways related to membrane biogenesis and fine-tuning of the balance between fast growing planktonic and sessile biofilm-associated lifestyles. We speculate that phase variation due to indels in intergenic homopolymeric tracts and adaptive changes engaging complex regulatory circuits with highly redundant c-di-GMP metabolic enzymes act in synergy, allowing *P. aeruginosa* to produce phenotypic diversity, maintain high versatility, and efficiently colonize new habitats.

## Supplementary information

Supplemental Material

Table S1

## Data Availability

The DNA sequencing data are deposited in the National Center for Biotechnology Information sequence read archive (http://www.ncbi.nlm.nih.gov/sra) under the BioProject accession no. PRJNA612188. The RNA sequencing data are deposited in the GEO repository (https://www.ncbi.nlm.nih.gov/geo) with the accession no. GSE146906. Custom scripts used to facilitate the processing of sequencing data can be provided upon request.
