# Supplementary material for: Evolution of Pseudomonas aeruginosa toward higher fitness under standard laboratory conditions
Source: ISME J. 2020 Dec 3;15(4):1165–77. doi: 10.1038/s41396-020-00841-6 (PMC8115180; doi:10.1038/s41396-020-00841-6)
Supplement: Supplementary file 1 — Supplemental Material [file 41396_2020_841_MOESM1_ESM.pdf]

Supplementary Information

Evolution of *Pseudomonas aeruginosa* towards higher fitness under standard laboratory conditions

Igor Grekov,[1,3] Janne Gesine Thöming,[2,3] Adrian Kordes,[2,4] Susanne Häussler[1,2,3,4]#

[1]Department of Molecular Bacteriology, Helmholtz Centre for Infection Research, Braunschweig, Germany

[2]Institute of Molecular Bacteriology, TWINCORE Centre for Experimental and Clinical Infection Research, Hannover, Germany

[3]Department of Clinical Microbiology, Copenhagen University Hospital – Rigshospitalet, Copenhagen, Denmark

[4]Cluster of Excellence RESIST (EXC 2155), Hannover Medical School, Hannover, Germany

Running Head: *Pseudomonas* fine-tunes lifestyle to promote growth

#Address correspondence to Susanne Häussler, Department of Molecular Bacteriology, Helmholtz Centre for Infection Research, Inhoffenstr. 7, Braunschweig, Germany; +4953161813000; Susanne.haeussler@helmholtz-hzi.de

Supplementary Note S1. Culture passaging

For passaging weak bottleneck (WBN) cultures, their endpoint OD_{600} was estimated using an EnSpire Multimode Plate Reader (PerkinElmer, Inc., Waltham, MA). The OD_{600} was then related to CFUs based on pre-experiments, which had established that, in hypermutator *P. aeruginosa*, OD_{600} = 1 corresponded to 10^9 CFUs/ml (0.978 ± 0.082 x 10^9 CFUs/ml, n = 3) and in hypermutator *S. enterica*, OD_{600} = 1 corresponded to 1.2 x 10^9 CFUs/ml (1.199 ± 0.042 x 10^9 CFUs/ml, n = 3). For passaging of strong bottleneck (SBN) cultures, two-fold serial dilutions of the overnight SBN cultures were prepared in round-bottom 96-well plates, 100 µl per well. The last wells with detectable bacterial growth were used to prepare new serial dilutions.

Supplementary Note S2. Indel analysis

In the detailed analysis of indels, all the comparisons of LBN and SBN groups were performed using strict grouping criteria: only the indels that occurred in one of the groups but not those that occurred both in LBN clones and SBN lines were included in the analysis. While comparing genes effected by upstream indels in P. aeruginosa LBN and SBN groups with genes effected by upstream indels in clinical isolates, indels were considered to be identical only if both the position, the length and the type (insertion or deletion) matched.

Supplementary Note S3. Preparation and sequencing of *P. aeruginosa* RNA libraries

All transcription analyses were performed in biological duplicates following planktonic growth in LB at 37°C with shaking at 180 rpm. To prepare a single biological replicate, 4 to 5 independent 5 ml cultures, with the starting concentration of 250 cells/ml, were cultivated to an OD600 between 0.4 – 0.6. Three cultures were pooled and mixed with an equal volume of RNAprotect prior to storing the bacterial pellets at -70°C. RNA was extracted using RNeasy Kit (Qiagen) combined with QIAshredder columns (Qiagen) as described previously (71). rRNA was depleted using a Ribo-Zero Bacteria Kit (Illumina). cDNA libraries were prepared using ScriptSeqTM v2 RNA-Seq Library Prep Kit (Illumina) according to the manufacturer's instructions. The cDNA libraries were depleted of primer dimers with an AMPure XP purification protocol and pooled prior to sequencing with an Illumina HiSeq 2500 in single end mode.

Supplementary Note S4. Fitness analysis

The growth of the starting and evolved cell lines was assessed by determining the optical density throughout the growth phase using the Bioscreen C (Oy Growth Curves Ab Ltd., Helsinki, Finland). Three technical replicates consisting of 100 µl cultures were inoculated with 250 cells and grown at 37°C with continuous shaking in Bioscreen 100-well plates in either LB medium, LB + 0.2% glucose or BM2 medium (40 mM K_2HPO_4, 22 mM KH_2PO_4) supplemented with 2 mM $MgSO_4$, 10 µM $FeSO_4$, 0.4% glucose and 0.5% CAS amino acids. Additionally, the growth of the *P. aeruginosa* starting cell line and three randomly selected WBN clones was estimated by assessing colony forming unit counts throughout the growth phase. One ml cultures were inoculated with 2500 cells and grown at 37°C with continuous shaking in LB medium in 13 ml plastic tubes. For each culture, every two hours, 10 µl were sampled, diluted and plated on an LB agar plate for subsequent counting of colony forming units.

Supplementary Note S5. Swimming motility

Technical triplicates of 1 µl bacterial suspensions with a final concentration of 4×10^{10} bacteria/ml were spotted on BM2-based 0.3% swimming agar with 0.4% glucose as the sole carbon and energy source. The bacteria were cultivated at 37° C in humid atmosphere; swimming zone diameter was recorded after 17 hours of incubation.

Supplementary Note S6. Virulence analysis

Ten larvae were infected with 20 µl of phosphate-buffered saline (PBS) containing 100 bacteria and incubated at 30°C. The ratio of dead larvae was assessed by inspection of cuticle melanisation and the loss of movement in response to stimulation.

Supplementary Note S7. Biofilm analysis by fluorescent confocal microscopy

Overnight cultures were adjusted to an OD600 of 0.002 and 100 µl of the bacterial suspensions were inoculated in sterile half-area, 96-well µClear microtiter plates (Greiner Bio-One, Kremsmünster, Austria). Microtiter plates were sealed and incubated statically at 37 °C in humid atmosphere for 24 hours. Afterwards, 60 µl of a solution containing Syto9 and propidium iodide (final concentrations of 2.1 µM and 12.5 µM respectively) of the LIVE/DEAD® BacLight™ Bacterial Viability Kit (Molecular Probes, Carlsbad, CA) was added to each well. After a second incubation, 48-hours-old biofilms were imaged using an automated confocal laser scanning microscope (SP8 System, Leica, Wetzlar, Germany) equipped with a HC PL APO 40x/1.10 W motCORR CS2 water immersion objective. Z-stacks composed of 20 focal planes with a z-step size of 3 µm were acquired in the center of each well. Images were analyzed with the customized software (Developer XD 64 version 2.0.4, Definiens AG) to quantify biofilm volume and thickness as previously described, except for manually setting a threshold for Syto9 to 25 instead of the automated detection.

Supplementary Note S8. Crystal violet assay

Overnight cultures were diluted to an OD600 of 0.02 in LB, inoculated in 100 µl in 96-well polyvinyl chloride plates (Costar), sealed with BREATHseal air-permeable membranes (Greiner Bio-One) and incubated under static conditions in a humid atmosphere at 37°C for 24 hours. Afterwards, the cultures were removed, the wells were washed with water and stained with 0.1% (wt/vol) crystal violet solution at room temperature. In 30 min, the staining solution was removed and the wells were again washed with water and air dried. The retained crystal violet was destained with 95% ethanol for 30 min at room temperature. For quantification of the surface-bound biomass, 125 µl of the solution was transferred to fresh polystyrene microtiter plates (Nunc), and the absorbance was measured at 550 nm using an EnSpire Multimode Plate Reader.

Supplementary Note S9. Scanning electron microscopy

Bacterial cultures at OD = 2.0 were fixed with aldehydes (final concentration 5% formaldehyde and 2% glutaraldehyde) and washed twice in TE buffer (20 mM TRIS, 1 mM EDTA, and pH 6.9). 50 µl were added to round, Poly-L-Lysine pretreated coverslips and incubated for 10 minutes at room temperature before a post fixation in TE buffer including 1% glutaraldehyde for another 10 minutes. Afterwards,

coverslips were washed twice in TE buffer and dehydrated on ice with a graded series of acetone (10%, 30%, 50%, 70%, and 90%) for 10 min each step, followed by two steps in 100% acetone at room temperature. Critical point drying with liquid CO2 was performed with the CPD 30 (Bal-Tec, Liechtenstein) and sputter coating (55s at 45 mA) with the SCD500 (Bal-Tec, Liechtenstein) to coat the coverslips (mounted onto aluminum stubs with carbon adhesive discs (Plano, Wetzlar)) with a thin gold palladium film. Samples were analyzed with a field emission scanning electron microscope Zeiss Merlin (Zeiss, Oberkochen) using the Everhart Thornley HESE2 detector and the inlens SE detector in a 25:75 ratio and with an acceleration voltage of 5 kV.

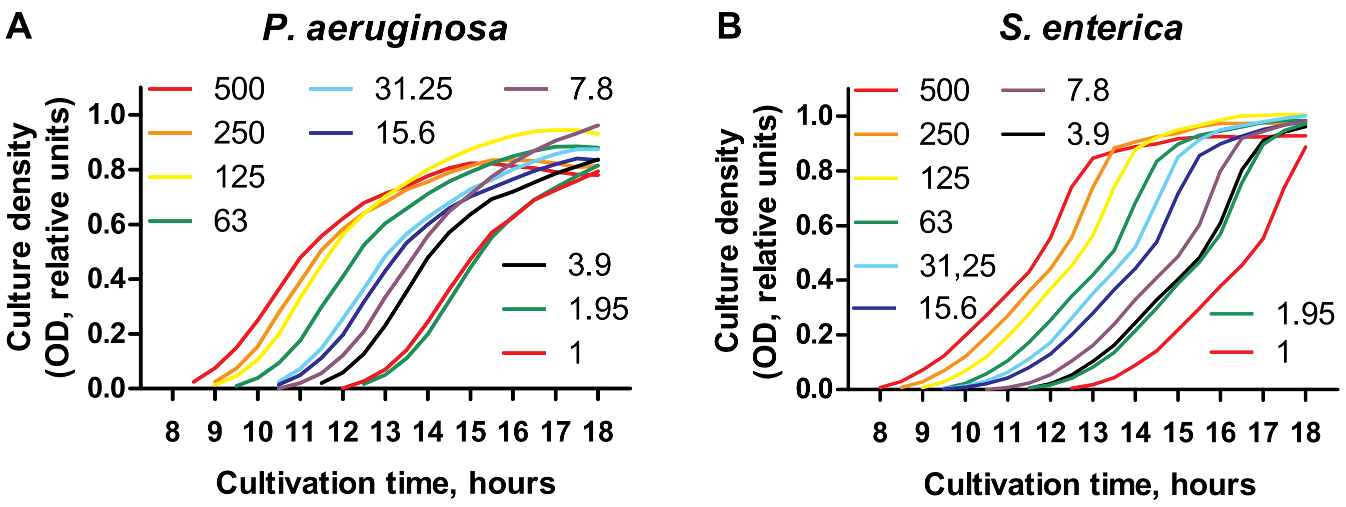

Fig. S1 Growth characteristics of the *P. aeruginosa mutS*::Tn and *S. enterica* ∆*mutS*. *P. aeruginosa mutS*::Tn starting cell line was grown in LB at 37°C (A) and *S. enterica* ∆*mutS* starting cell line in LB + 0.2% glucose at 30°C (B). Each curve describes the growth of a culture that has been inoculated with the indicated number of bacteria. *S. enterica* graph is a representative of five independent experiments and *P. aeruginosa* graph is a representative of three independent experiments.

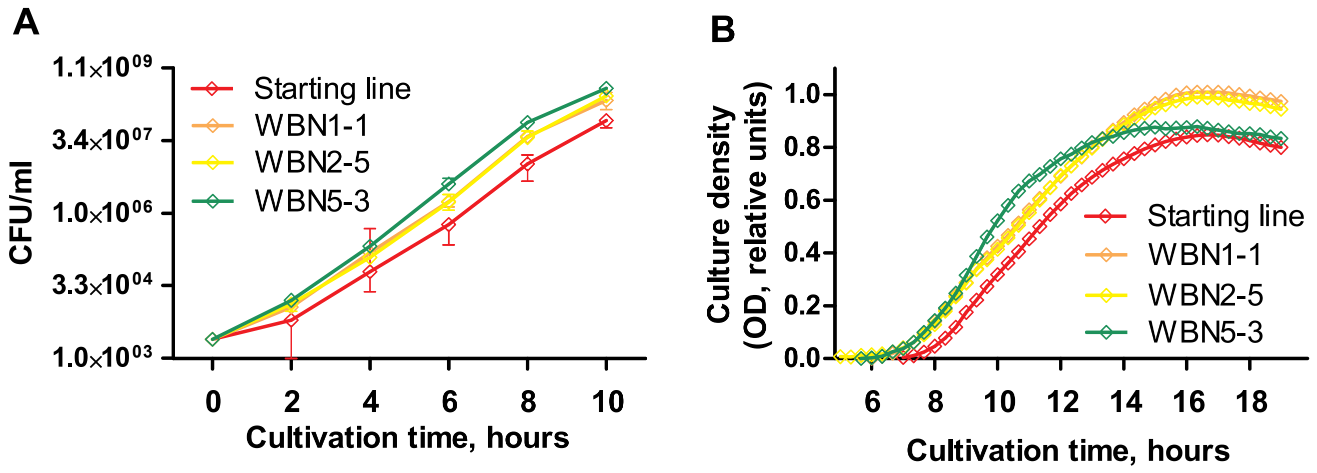

Fig. S2 Growth of the evolved *P. aeruginosa* clones according to colony forming units/ml. The growth of the *P. aeruginosa* starting cell line and three randomly selected WBN clones (WBN1-1, WBN2-5 and WBN5-3) in LB medium at 37°C was estimated by measuring colony forming units/ml (A). The data show median and range based on three biological replicates. For comparison, the growth of the same clones as previously determined according to OD_{600} is shown (B).

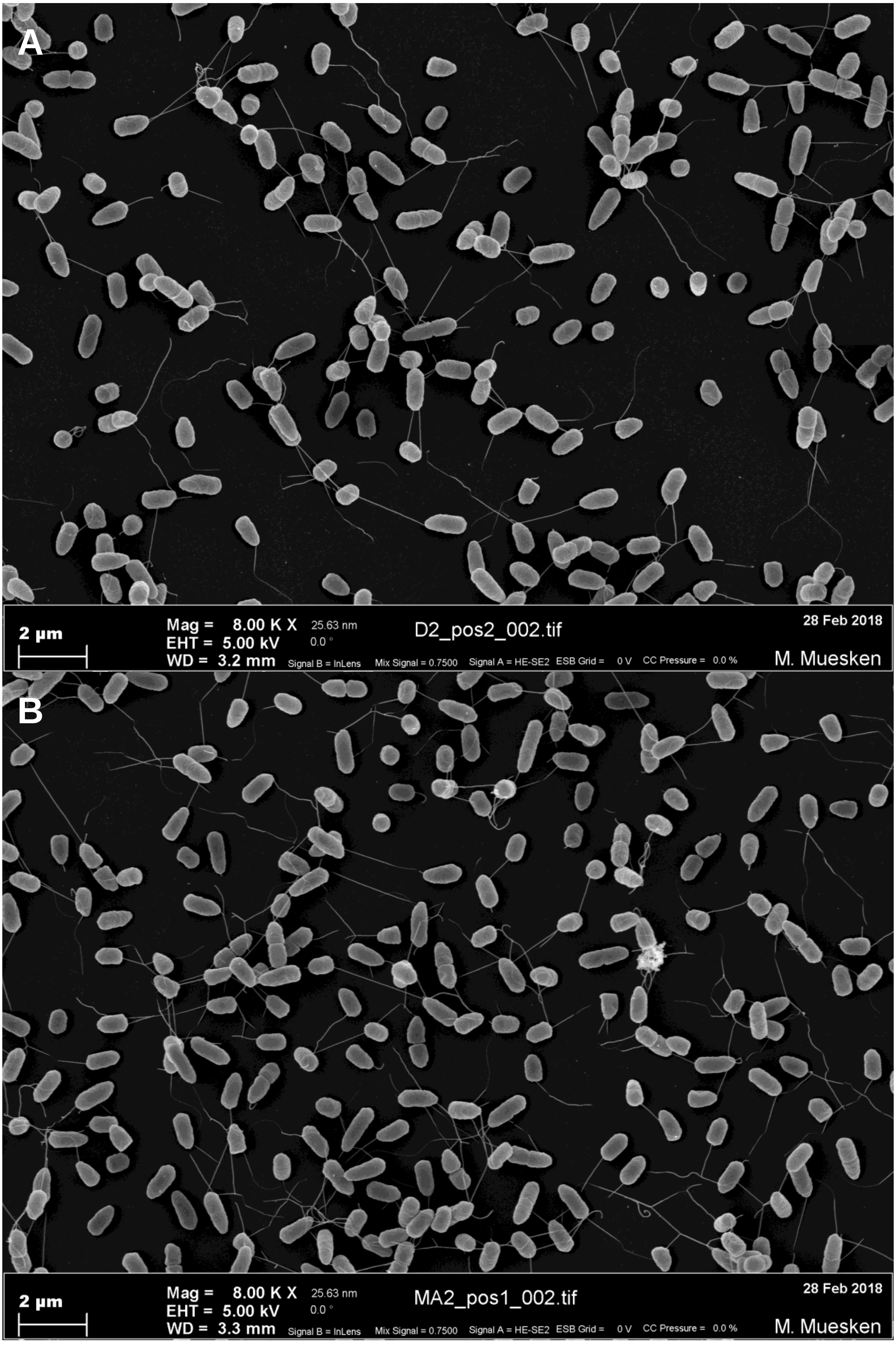

Fig. S3 Bacterial cell size. *P. aeruginosa* starting line cells (A) and a randomly selected WBN clone cells (B) were sampled at OD = 2 and their size was compared using scanning electron microscopy (Supplementary Note S9). We observed no differences in cell size between the starting line and a WBN clone.

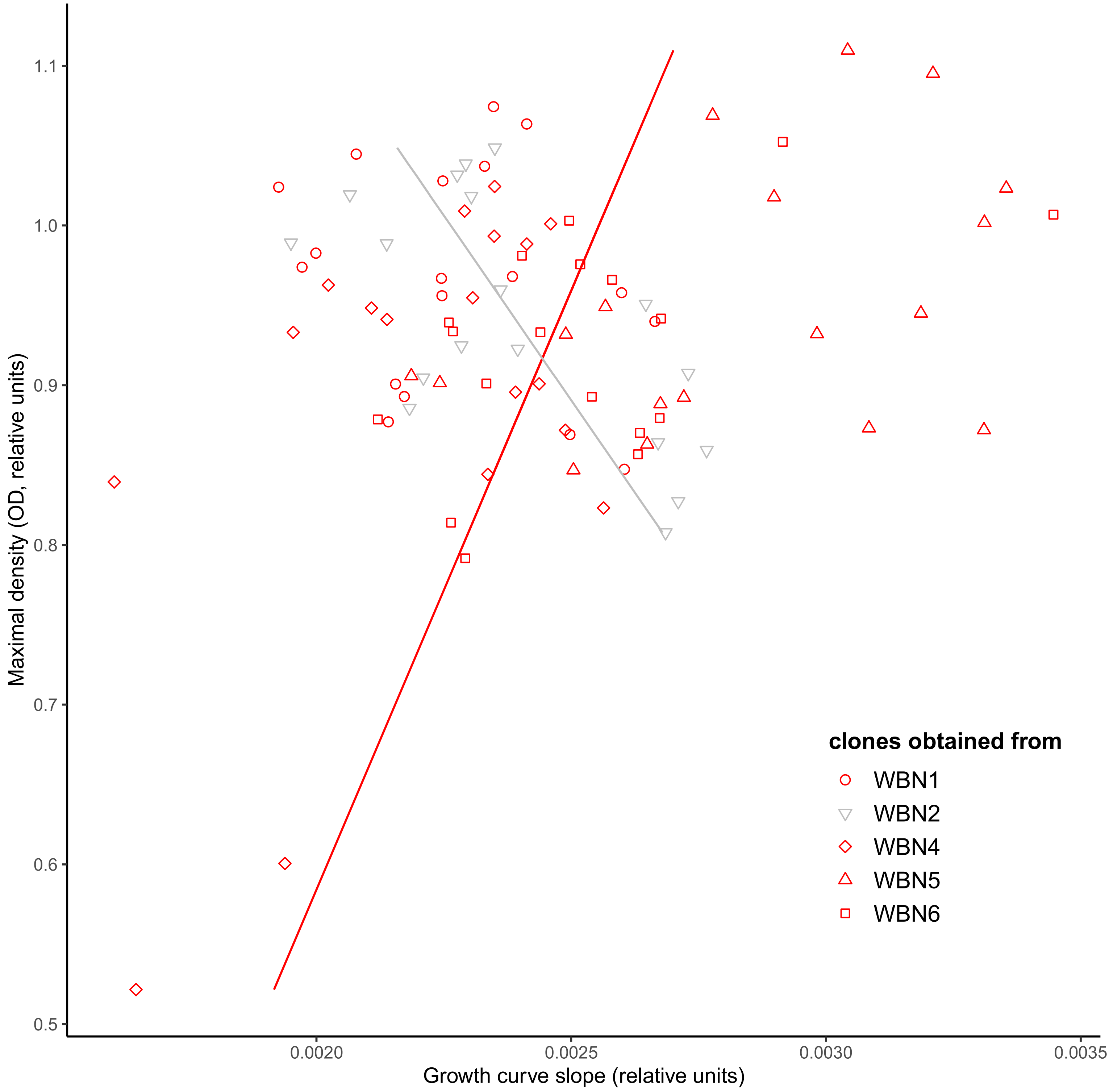

Fig. S4 Correlation of growth yield and growth rate. The relationship between growth rate and maximal density across 6 clones from overall 5 WBN cell lines was analyzed using linear regression (3 independent experiments, three technical replicates). Four of five WBN cell lines (red symbols) show significant positive correlation between growth rate and maximal culture density (red line, P = 0.004344). In the remaining WBN cell line (gray symbols) there was a negative correlation between growth rate and maximal culture density (gray line, P = 0.003765).

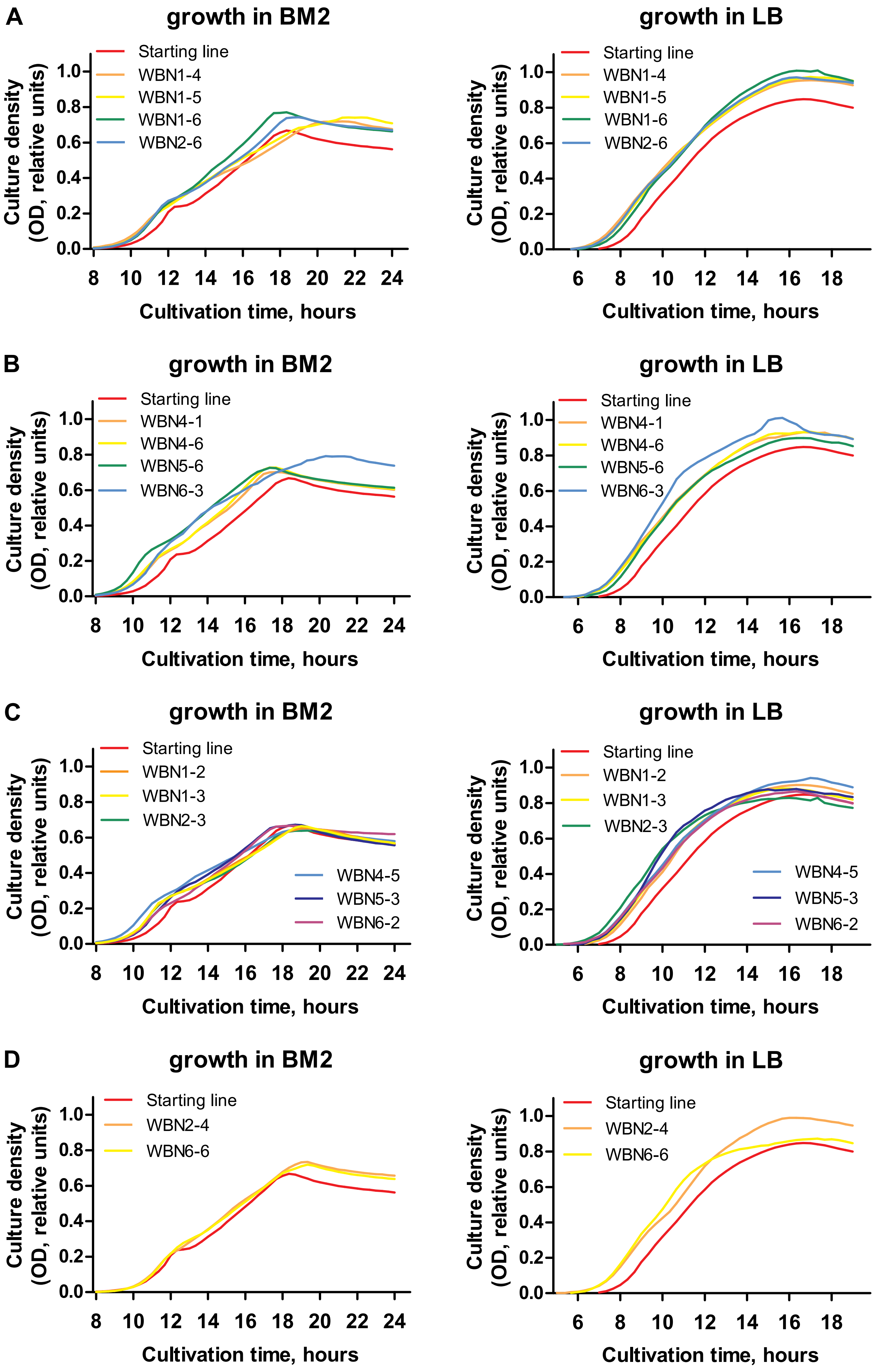
A
growth in BM2
Starting line
WBN1-4
WBN1-5
WBN1-6
WBN2-6
Culture density (OD, relative units)
Cultivation time, hours
growth in LB
Starting line
WBN1-4
WBN1-5
WBN1-6
WBN2-6
Culture density (OD, relative units)
Cultivation time, hours
B
growth in BM2
Starting line
WBN4-1
WBN4-6
WBN5-6
WBN6-3
Culture density (OD, relative units)
Cultivation time, hours
growth in LB
Starting line
WBN4-1
WBN4-6
WBN5-6
WBN6-3
Culture density (OD, relative units)
Cultivation time, hours
C
growth in BM2
Starting line
WBN1-2
WBN1-3
WBN2-3
WBN4-5
WBN5-3
WBN6-2
Culture density (OD, relative units)
Cultivation time, hours
growth in LB
Starting line
WBN1-2
WBN1-3
WBN2-3
WBN4-5
WBN5-3
WBN6-2
Culture density (OD, relative units)
Cultivation time, hours
D
growth in BM2
Starting line
WBN2-4
WBN6-6
Culture density (OD, relative units)
Cultivation time, hours
growth in LB
Starting line
WBN2-4
WBN6-6
Culture density (OD, relative units)
Cultivation time, hours

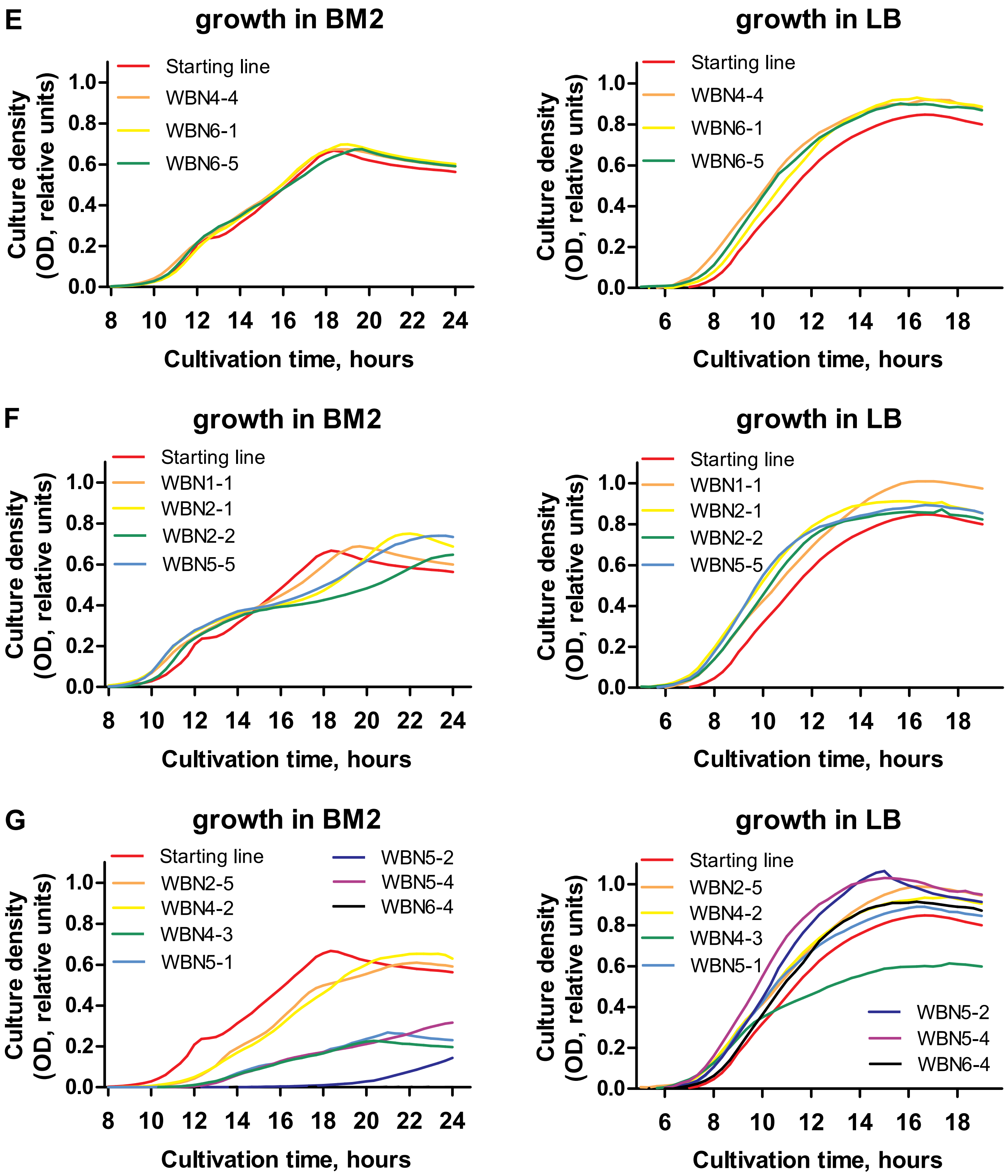

Fig. S5 Growth characteristics of the evolved *P. aeruginosa* clones. The growth of the *P. aeruginosa* starting cell line and of the 30 WBN clones (6 from each of the 5 WBN cell lines, Fig. 1) in BM2 medium at 37°C was recorded in 3 independent experiments with 3 technical replicates. Eight WBN clones surpassed the starting cell line in all three parameters tested: shortening of lag phase, overall growth rate and maximal OD600 values (A,B). Six WBN clones displayed a shorter lag phase, but the same growth rate and maximal OD600 (C). Two WBN clones showed comparable growth in the lag phase, a comparable overall growth rate, but higher maximal OD600 values (D). Three WBN clones displayed growth parameters that were similar to those of the starting line (E). Four WBN clones showed a mixed phenotype exhibiting a shorter lag phase, a similar or higher maximal OD600 but a lower overall growth rate (F). Finally, seven WBN clones displayed a variety of growth defects, which ranged from no growth in BM2 medium to a longer lag phase in comparison to the starting line (G).

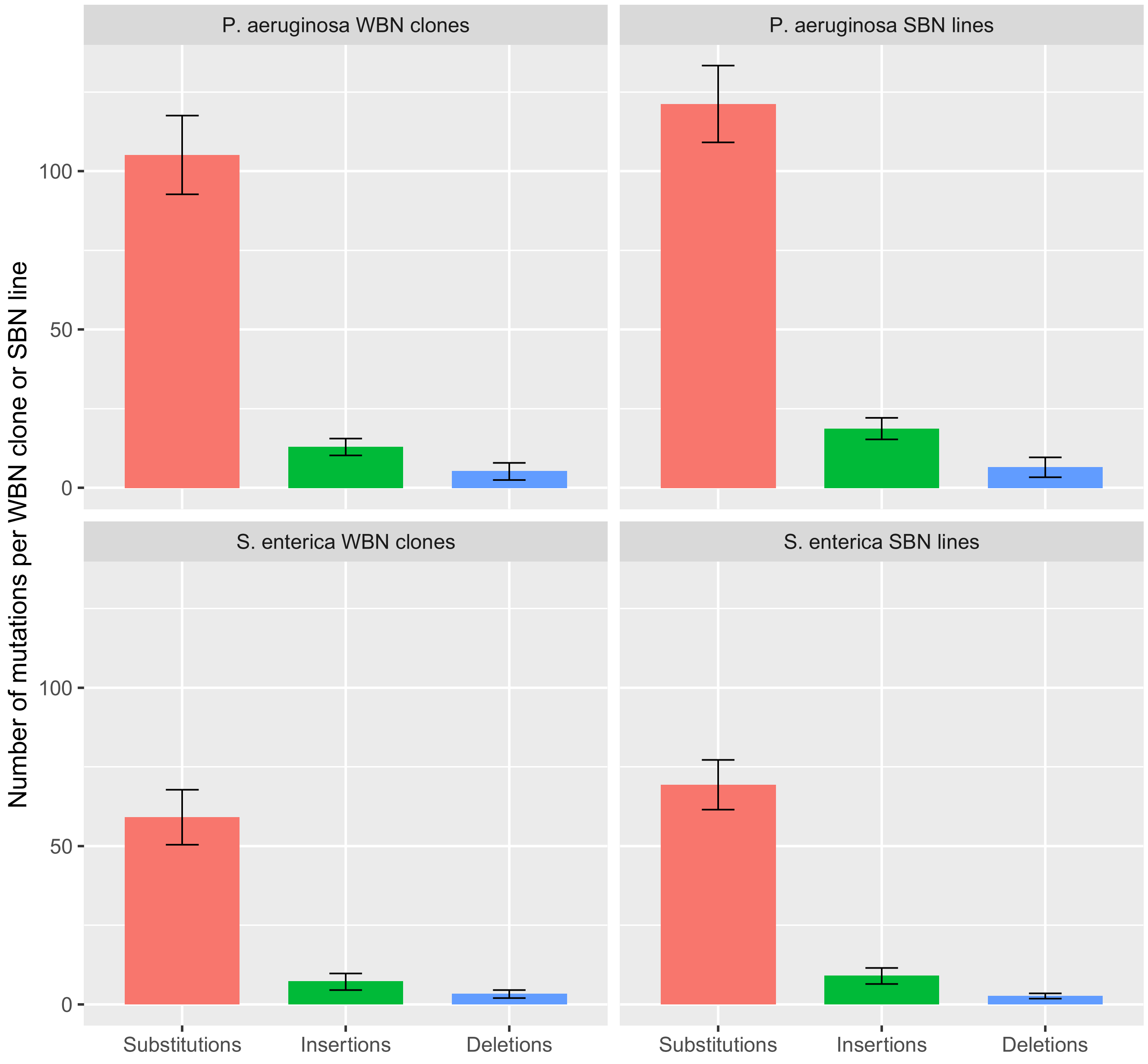

Fig. S6 Types of mutations accumulated. Mutations were counted for 30 P. aeruginosa WBN clones, 6 P. aeruginosa SBN lines, 29 S. enterica WBN clones and 6 S. enterica SBN lines. The data represent mean and standard deviation.

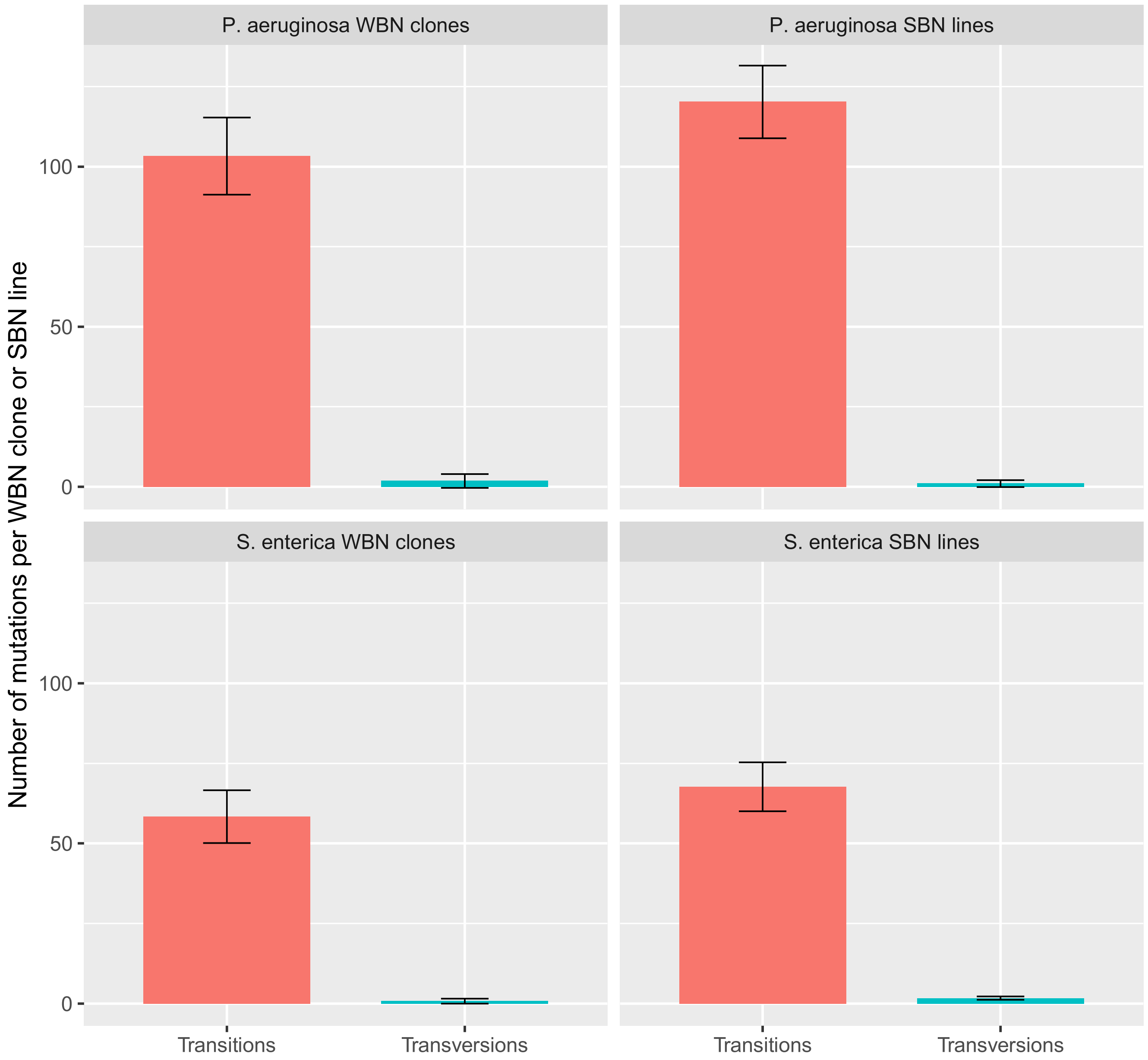

Fig. S7 Types of nucleotide substitutions. Mutations were counted for 30 P. aeruginosa WBN clones, 6 P. aeruginosa SBN lines, 29 S. enterica WBN clones and 6 S. enterica SBN lines. The data represent mean and standard deviation.

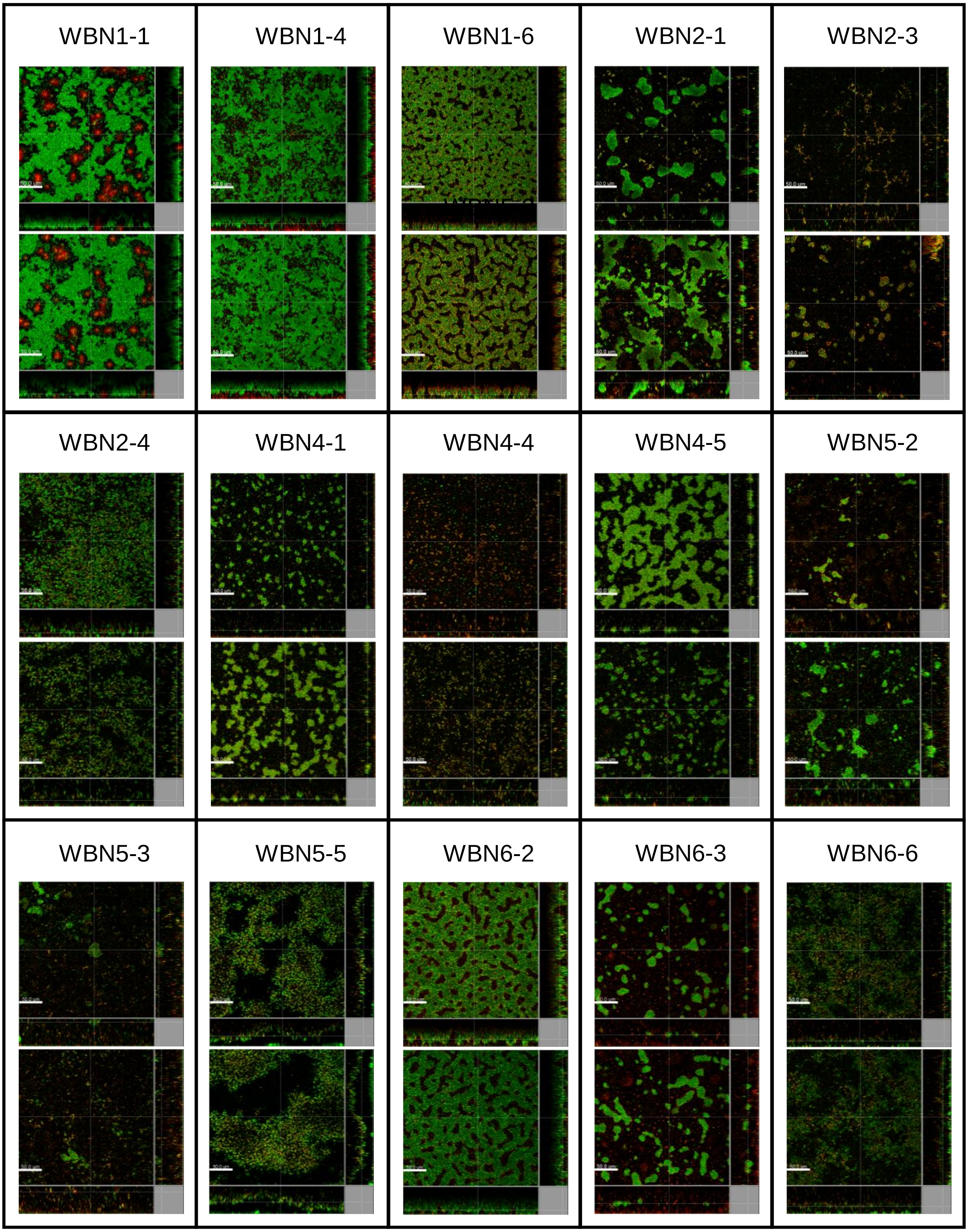

Fig. S8 The fluorescent confocal microscopy images of the biofilms formed by *P. aeruginosa* WBN clones. The images of biological duplicates of 15 WBN clones for which transcriptional profiles have been recorded are shown. Green color represents the living cells (Syto9 staining) and red color represents the dead cells (propidium iodide staining).
